# Establishment and external validation of a nomogram for predicting 28-day mortality in patients with skull fracture

**DOI:** 10.3389/fneur.2023.1338545

**Published:** 2024-01-12

**Authors:** Jia Tang, Zhenguang Zhong, Muyesai Nijiati, Changdong Wu

**Affiliations:** ^1^Graduate School of Xinjiang Medical University, Ürümqi, China; ^2^Department of Bioengineering, Imperial College London, London, United Kingdom; ^3^Xinjiang Emergency Center, People's Hospital of Xinjiang Uygur Autonomous Region, Ürümqi, China

**Keywords:** skull fracture, head trauma, prognosis, nomogram, prediction model

## Abstract

**Background:**

Skull fracture can lead to significant morbidity and mortality, yet the development of effective predictive tools has remained a challenge. This study aimed to establish and validate a nomogram to evaluate the 28-day mortality risk among patients with skull fracture.

**Materials and methods:**

Data extracted from the Medical Information Mart for Intensive Care (MIMIC) database were utilized as the training set, while data from the eICU Collaborative Research Database were employed as the external validation set. This nomogram was developed using univariate Cox regression, best subset regression (BSR), and the least absolute shrinkage and selection operator (LASSO) methods. Subsequently, backward stepwise multivariable Cox regression was employed to refine predictor selection. Variance inflation factor (VIF), akaike information criterion (AIC), area under the receiver operating characteristic curve (AUC), concordance index (C-index), calibration curve, and decision curve analysis (DCA) were used to assess the model's performance.

**Results:**

A total of 1,527 adult patients with skull fracture were enrolled for this analysis. The predictive factors in the final nomogram included age, temperature, serum sodium, mechanical ventilation, vasoactive agent, mannitol, extradural hematoma, loss of consciousness and Glasgow Coma Scale score. The AUC of our nomogram was 0.857, and C-index value was 0.832. After external validation, the model maintained an AUC of 0.853 and a C-index of 0.829. Furthermore, it showed good calibration with a low Brier score of 0.091 in the training set and 0.093 in the external validation set. DCA in both sets revealed that our model was clinically useful.

**Conclusion:**

A nomogram incorporating nine features was constructed, with a good ability in predicting 28-day mortality in patients with skull fracture.

## Introduction

A skull fracture denotes the breakage of one or more bones within the skull vault or base, typically resulting from road traffic accidents, falls, or acts of violence. Head injuries are more commonly observed in middle- and low-income countries, particularly in the context of road traffic collisions (1). In a retrospective study involving 2,254 cases of head trauma caused by assault, approximately one-third of the patients were found to suffer from a skull fracture (2). The most prevalent skull fracture occurs at the parietal bone, followed by the temporal, occipital, and frontal bones (3). Unlike fractures in other body parts, skull fracture, in the absence of injury to vital structures like brain tissue, are not immediately life-threatening and often heal spontaneously. However, severe skull fracture can lead to concomitant trauma-related intracranial injuries, such as cerebral edema, subarachnoid hemorrhage, and cerebral lacerations, resulting in prolonged hospitalization and increased treatment costs. Traumatic intracranial hemorrhage (tICH) is a significant source of morbidity and mortality in trauma patients. Subdural hematoma (SDH), occurring in 11% to 49% of patients after traumatic brain injury (TBI), is commonly associated with an all-cause mortality rate of 14.2% to 53.8% within a five-year period (4).

Compared to TBI, skull fractures can be diagnosed both earlier and more easily, offering a more direct point for assessment and intervention. The development of a severity scoring system that accounts for mortality risk is imperative to accurately evaluate the prognosis and guide treatment strategies for patients with skull fracture. It not only aids in the early identification of patients with potentially fatal skull fractures but also optimizes resource distribution and patient management, particularly in emergency medical settings.

In recent years, there has been an upsurge in studies focusing on nomograms pertaining to head injuries. Nomograms offer an intuitive graphical representation, enhancing the comprehension and application of complex mathematical models and statistical methods. Healthcare professionals can expeditiously calculate individual risks by directly interpreting the variables and scales depicted in the chart (5). Chen et al. (6) formulated a predictive nomogram for assessing mortality in TBI patients, incorporating eight features (mannitol use, mechanical ventilation, vasopressor use, international normalized ratio, urea nitrogen, respiratory rate, and cerebrovascular disease). Lin et al. (7) developed a model to predict post-traumatic epilepsy (PTE) after cerebral contusions using seven variables [contusion site, chronic alcohol use, contusion volume, skull fracture, subdural hematoma (SDH), Glasgow coma scale (GCS) score, and non-late post-traumatic seizure], and the model exhibited excellent performance with a C index exceeding 0.9.

However, to the best of our knowledge, there currently exists no predictive model for assessing 28-day mortality in critically ill patients diagnosed with skull fracture. This study utilized two large public databases, namely the Medical Information Mart for Intensive Care (MIMIC) database and eICU Collaborative Research Database (eICU-CRD), to systematically screen predictive factors for 28-day mortality of critically ill patients with skull fracture. Subsequently we constructed a nomogram and ensured its validation. This model can be utilized by medical practitioners to enhance clinical decision-making, accurately anticipate mortality rates, and reduce uncertainty.

## Materials and methods

### Data source

Our data were sourced from the MIMIC-IV database (version 2.0), MIMIC-III Clinical Database CareVue subset (version 1.4) and eICU-CRD (version 2.0). MIMIC is a substantial, single-center, openly accessible database. It encompasses data from 2001 to 2019 within the intensive care units (ICUs) at Beth Israel Deaconess Medical Center. The eICU database is a multi-center database comprising health data associated with over 200,000 admissions to ICUs across the United States between 2014 and 2015. Rigorous de-identification measures have been applied to patient identity and hospital information in both databases. After successfully completing the training courses stipulated by the National Institutes of Health (NIH) and passing the relevant examination, we were granted permission to extract data. The first author of this study, Jia Tang, has completed the Collaborative Institutional Training Initiative (CITI) program courses and executed the data use agreement, thereby obtaining full access to the aforementioned two databases (record ID: 52759164).

### Research population

All patients with skull fracture from the two databases were included in this study. For patients with multiple ICU admission, only data from the initial ICU admission were considered. None of the participants in our research were minors, and we exclusively enrolled individuals with a minimum ICU duration of 24 h. The Structured Query Language (SQL) queries used for the selection of patients with skull fracture are presented in Supplementary Table S1.

### Feature extraction

We extracted the following data: age, sex, race, vital signs, laboratory data and Glagow Coma Scale (GCS) within 24 h after ICU admission (heart rate, systolic blood pressure, diastolic blood pressure, respiratory rate, temperature, hemoglobin, white blood cell count, platelet, prothrombin time, sodium, potassium, bicarbonate, serum creatinine); therapeutic interventions in the first 24 h (mechanical ventilation, vasoactive agent, furosemide, mannitol, human serum albumin, antibiotics); surgery for fracture; comorbidities (hypertension, chronic obstructive pulmonary disease, congestive heart failure, liver cirrhosis, cancer), fracture site (vault, base), open fracture, closed fracture, hemorrhage (subarachnoid hemorrhage, subdural hematoma, extradural hematoma), cerebral laceration and contusion, concussion, loss of consciousness. Additionally, we incorporated the acute physiology score III (APSIII), a widely employed tool for accessing disease severity. In this study, vasoactive agents included dopamine, dobutamine, epinephrine, norepinephrine, phenylephrine, vasopressin, and milrinone. Antibiotic users did not encompass those who administered antibiotics nasally or ocularly, nor did they include individuals using antibiotic creams or gels. Surgical data pertained to whether surgical operations were conducted during hospitalization due to skull fracture. The primary endpoint for this research was mortality within 28 days following ICU admission. Any variable with missing data exceeding 20% was removed from the analysis. Missing values for all variables were imputed by multiple imputation method (8). All requested information was obtained using Navicat 16 for PostgreSQL through Structured Query Language (SQL).

### Statistical analysis

Data from the MIMIC-IV databse and MIMIC-III carevue subset served as the training set and underwent 10-fold cross-validation, while data from eICU-CRD were used for external validation. The Shapiro-Wilk normality test was applied to all continuous variables. For normally distributed variables, the mean and standard deviation (SD) were utilized, while non-normally distributed variables were represented as the median and interquartile range (IQR). Categorical variables were expressed as numerical values and percentages (%). The distinction between two groups was evaluated using the *t* test or Wilcoxon rank-sum test for continuous variables and the chi-square test for categorical variables. Statistical significance was determined with a two-sided *P* < 0.05. R software (version 4.3.1) and MedCalc (version 22.009) were employed to perform the statistical analyses.

### Model development phase

In the initial screening for significant prognostic factors, we employed three methods: univariate Cox regression, best subset regression (BSR), and least absolute shrinkage and selection operator (LASSO). In the univariate Cox model, factors with a *P* < 0.05 were included in subsequent analysis. The BSR method evaluated all possible variable combinations and selected final variables based on the maximum value of adjusted R^2^. LASSO regression determined variable selection based on the lambda.1se value. Subsequently, each of the three models underwent backward stepwise multivariate Cox regression for secondary screening of independently significant factors. Ultimately, the akaike information criterion (AIC) and receiver operating characteristic (ROC) curves were employed to identify the optimal model, leading to the construction of a nomogram. Any variable that contradicted established clinical knowledge was eliminated. We calculated the variance inflation factor (VIF) to ensure no collinearity among the selected covariates (collinearity was considered when VIF > 4.0). Additionally, we checked whether the nomogram adhered to the “10 EPV” guideline (9), which stipulates that the number of positive samples should be at least ten times the number of predictive variables.

For internal validation section, we employed 200 rounds of 10-fold cross validation to assess the model performance. The discrimination of the nomogram was assessed using the area under the receiver operating curve (AUC) and concordance index (C-index). The AUCs of the three models were compared using the DeLong's test. We assessed the consistency between the nomogram-predicted and actual outcomes using a calibration curve generated through bootstrapped resampling (1,000 iterations) and the Brier score (ranging from 0 to 1, with 0 indicating perfect calibration). Decision curve analysis (DCA) was conducted to exhibit the clinical usefulness of the nomogram. Using the nomogram, we calculated total points for each patient and performed risk stratification using the X-tile (version 3.6.1) software (10). This categorization divided patients into low, mid, and high-risk groups. The survival outcomes of three groups were analyzed using Kaplan-Meier survival curves and evaluated by log-rank test.

## Results

### Patient characteristics

**Figure 1:**
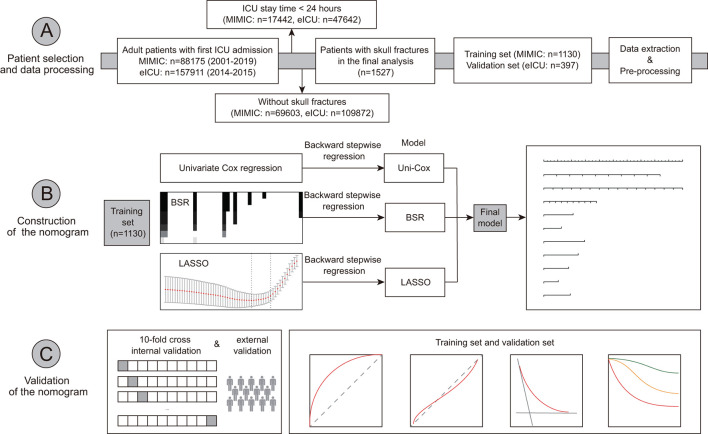
Overview of the research workflow.

This study included a total of 1,527 records of critically ill patients with skull fractures, comprising 1,130 patients from the MIMIC database and 397 patients from the eICU database (Figure 1). Supplementary Figures S1, S2 showed that the proportion of all missing values in two groups was less than 20%. No statistical significance was observed in the data, either before or after imputation (Supplementary Tables S2, S3). Moreover, there was no statistically significant difference between training set and external validation set in terms of heart rate, respiratory rate, WBC, platelet, sodium, serum creatinine, surgery for fracture, COPD, liver cirrhosis, cancer, open fracture, and cerebral laceration and contusion (Table 1). Patients in the training set had older age [52.16 (95% CI: 31.18–67.89) vs. 44.00 (95% CI: 28.00–65.00), *P* = 0.001], higher body temperature [36.83 (95% CI: 36.33–37.22) vs. 36.70 (95% CI: 36.28–37.00), *P* < 0.001], a higher mechanical ventilation use rate [661 (58.5%) vs. 201 (50.6%), *P* = 0.008], a higher vasoactive agent use rate [218 (19.3%) vs. 37 (9.3%), *P* < 0.001], a higher mannitol use rate [138 (12.2%) vs. 31 (7.8%), *P* = 0.021], a higher extradural hematoma rate [566 (50.1%) vs. 35 (8.8%), *P* < 0.001] than those in the external validation set. The GCS score in the training set was also higher.

**Table 1:** Demographic and clinical characteristics of the training and validation sets.

**Variables**	**Total**	**Training set**	**Validation set**	***P* value**
	***n*** = **1,527**	***n*** = **1,130**	***n*** = **397**	
Age^a^	50.43 [30.19, 67.64]	52.16 [31.18, 67.89]	44.00 [28.00, 65.00]	0.001
Female^b^	424 (27.8)	330 (29.2)	94 (23.7)	0.040
**Race** ^b^				< 0.001
White	997 (65.3)	677 (59.9)	320 (80.6)	
Black	55 (3.6)	26 (2.3)	29 (7.3)	
Others	475 (31.1)	427 (37.8)	48 (12.1)	
**Vital signs** ^a^				
Heart rate, bpm	86.00 [73.00, 100.00]	86.00 [73.00, 99.00]	86.00 [71.00, 102.00]	0.829
SBP, mmHg	131.00 [115.00, 146.00]	131.00 [115.00, 144.00]	134.00 [116.00, 148.00]	0.030
DBP, mmHg	71.00 [61.00, 83.00]	70.00 [60.00, 81.00]	77.00 [65.00, 89.00]	< 0.001
Respiratory rate, bpm	18.00 [15.00, 21.00]	18.00 [15.00, 21.00]	18.00 [16.00, 21.00]	0.928
Temperature, °C	36.78 [36.31, 37.21]	36.83 [36.33, 37.22]	36.70 [36.28, 37.00]	< 0.001
**Laboratory results** ^a^				
Hb, g/dL	13.20 [11.60, 14.40]	12.90 [11.40, 14.20]	13.60 [12.10, 14.80]	< 0.001
WBC, 10^9^/L	13.20 [9.70, 17.50]	13.30 [9.80, 17.80]	12.80 [9.30, 17.00]	0.128
Platelet, 10^9^/L	225.00 [182.00, 277.00]	226.00 [182.00, 280.00]	223.00 [182.00, 264.00]	0.278
PT, s	12.80 [11.70, 13.90]	12.60 [11.60, 13.50]	13.50 [12.20, 14.50]	< 0.001
Sodium, mmol/L	140.00 [137.00, 142.00]	140.00 [137.00, 142.00]	139.00 [137.00, 141.00]	0.163
Potassium, mmol/L	3.90 [3.60, 4.30]	3.90 [3.60, 4.30]	3.80 [3.50, 4.20]	< 0.001
Bicarbonate, mmol/L	23.00 [20.00, 25.00]	23.00 [20.00, 25.00]	23.00 [21.00, 26.00]	0.019
SCr, mg/dL	0.90 [0.70, 1.10]	0.90 [0.70, 1.10]	0.90 [0.73, 1.10]	0.108
**Therapeutic interventions** ^b^				
MV	862 (56.5)	661 (58.5)	201 (50.6)	0.008
Vasoactive agent	255 (16.7)	218 (19.3)	37 (9.3)	< 0.001
Furosemide	57 (3.7)	54 (4.8)	3 (0.8)	< 0.001
Mannitol	169 (11.1)	138 (12.2)	31 (7.8)	0.021
HSA	36 (2.4)	34 (3.0)	2 (0.5)	0.008
Antibiotics	708 (46.4)	651 (57.6)	57 (14.4)	< 0.001
Surgery for fracture^b^	167 (10.9)	133 (11.8)	34 (8.6)	0.095
**Comorbidities** ^b^				
Hypertension	404 (26.5)	325 (28.8)	79 (19.9)	0.001
COPD	25 (1.6)	17 (1.5)	8 (2.0)	0.646
CHF	73 (4.8)	10 (2.5)	63 (5.6)	0.020
Liver cirrhosis	27 (1.8)	23 (2.0)	4 (1.0)	0.265
Cancer	37 (2.4)	24 (2.1)	13 (3.3)	0.274
**Fracture site** ^b^				
Vault of skull	342 (22.4)	253 (22.4)	89 (22.4)	1.000
Base of skull	851 (55.7)	783 (69.3)	68 (17.1)	< 0.001
**Fracture feature** ^b^				
Open fracture	106 (6.9)	83 (7.3)	23 (5.8)	0.352
Closed fracture	1,101 (72.1)	1,039 (91.9)	62 (15.6)	< 0.001
**Hemorrhage** ^**b**^				
Subarachnoid hemorrhage	892 (58.4)	716 (63.4)	176 (44.3)	< 0.001
Subdural hematoma	908 (59.5)	730 (64.6)	178 (44.8)	< 0.001
Extradural hematoma	601 (39.4)	566 (50.1)	35 (8.8)	< 0.001
Cerebral laceration and contusion^b^	181 (11.9)	137 (12.1)	44 (11.1)	0.644
Concussion^b^	38 (2.5)	22 (1.9)	16 (4.0)	0.035
Loss of consciousness^b^	787 (51.5)	729 (64.5)	58 (14.6)	< 0.001
GCS (point)^a^	13.00 [7.00, 15.00]	13.00 [9.00, 15.00]	8.00 [3.00, 14.00]	< 0.001

^a^Expressed as median [IQR]; ^b^expressed as n (%).

IQR, interquartile range; bpm, beats/breaths per minute; SBP, systolic blood pressure; DBP, diastolic blood pressure; Hb, hemoglobin; WBC, white blood cell count; PT, prothrombin time; SCr, serum creatinine; MV, mechanical ventilation; HSA, human serum albumin; COPD, chronic obstructive pulmonary disease; CHF, congestive heart failure; GCS, Glasgow Coma Scale.

### Construction of the nomogram

**Figure 2:**
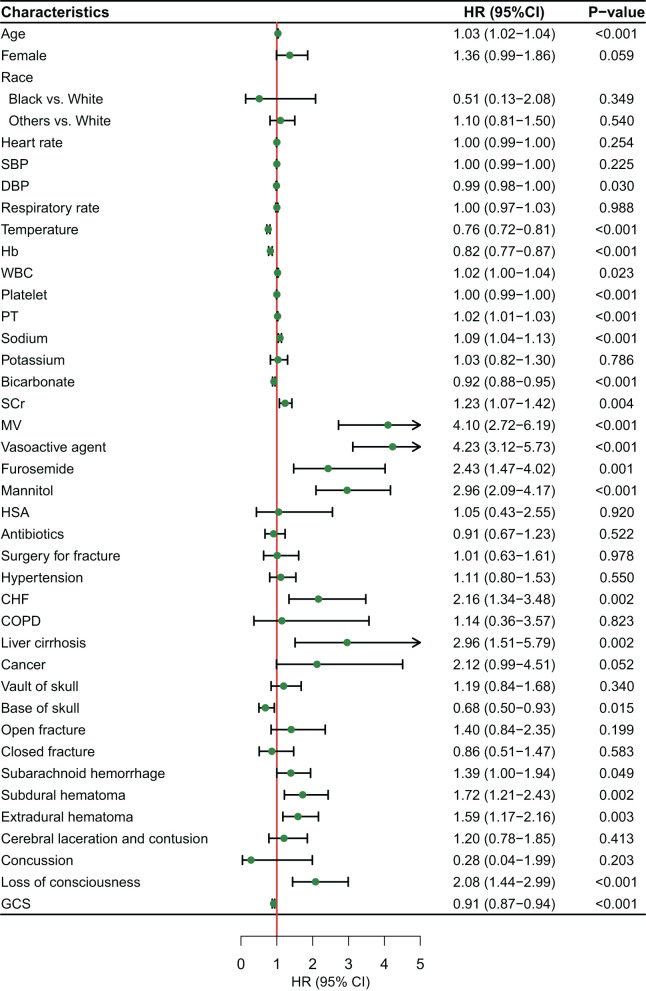
Features selection by univariate Cox regression.

**Figure 3:**
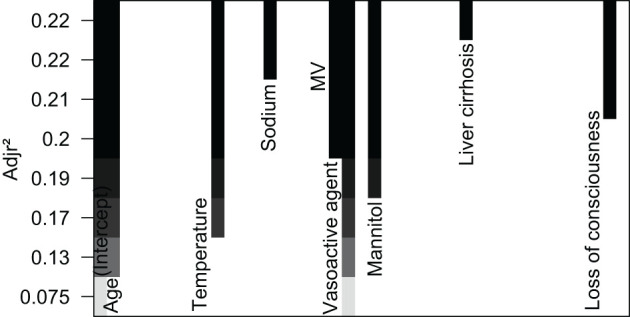
Features selection by BSR.

**Figure 4:**
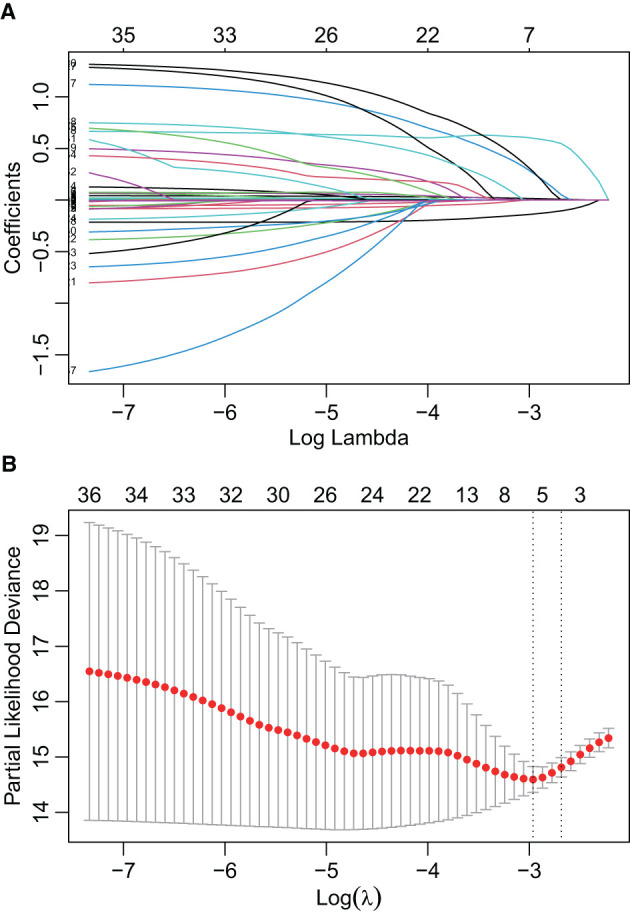
Features selection by LASSO. **(A)** LASSO coefficient profiles of the candidate predictors. **(B)** 10-fold cross-validation for tuning parameter selection in the LASSO model.

**Figure 5:**
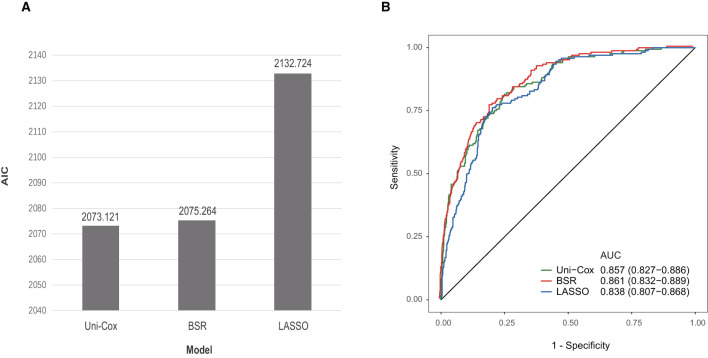
Comparison among three models. **(A)** Comparison of AIC values among three models. **(B)** Comparison of AUC values among three models.

**Figure 6:**
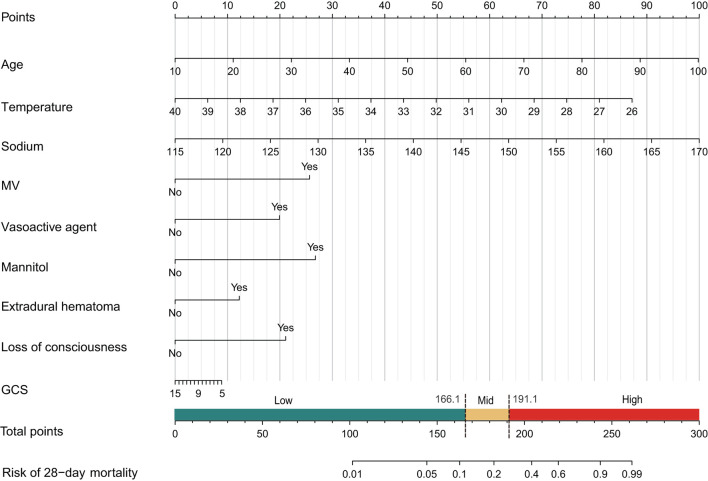
Nomogram for predicting the risk of 28-day mortality in patients with skull fracture.

In the initial stage, 22 significant (defined as factors with P values less than 0.05) features (age, DBP, temperature, Hb, WBC, bicarbonate, platelet, PT, sodium, liver cirrhosis, SCr, MV, vasoactive agent, furosemide, mannitol, congestive heart failure, base fracture, subarachnoid hemorrhage, subdural hematoma, extradural hematoma, loss of consciousness, and GCS) were selected through univariate Cox hazard analysis (Figure 2). Using the maximum value of adjusted R^2^ (value: 8) from BSR, we identified eight variables (age, temperature, MV, vasoactive agent, mannitol, sodium, liver cirrhosis, and loss of consciousness) (Figure 3). Additionally, four variables (age, temperature, mv, and vasoactive agent) were selected through LASSO regression using the lambda.1se value (Figure 4). Subsequently, we conducted a backward stepwise multivariable Cox regression analysis on the variables of each model to identify factors with *P* < 0.01 (Table 2). Considering the importance of GCS for neurological disorders and its ease of accessibility, we have additionally added this parameter to each model, even though it was eliminated in the statistical screening process. We compared the AIC and AUC values among three models (Figure 5). In the uni-Cox model (nine variables: age, temperature, sodium, MV, vasoactive agent, mannitol, extradural hematoma, loss of consciousness and GCS score), the AIC was 2073.121 and AUC was 0.857 (95% CI: 0.827–0.886); in the BSR model (nine variables: age, temperature, sodium, MV, vasoactive agent, mannitol, liver cirrhosis, loss of consciousness and GCS score), the AIC was 2075.264 and AUC was 0.861 (95% CI: 0.832–0.889); in the LASSO model (five variables: age, temperature, mv, vasoactive agent, and GCS), the AIC was 2132.724 and AUC was 0.838 (95% CI: 0.807–0.868). The AUC of the uni-Cox model was significantly different from the LASSO model (DeLong's test: *P* = 0.019), but there was no significant difference between the uni-Cox model and BSR model (DeLong's test: *P* = 0.328). Moreover, the uni-Cox model achieved the highest sensitivity at 81.5%, with the BSR model delivering the highest specificity at 80.4% (Supplementary Table S4). As a result, the first model with nine factors (age, temperature, sodium, MV, vasoactive agent, mannitol, extradural hematoma, loss of consciousness and GCS score) were included in the nomogram due to its lowest AIC and highest AUC among three models (Figure 6).

**Table 2:** Final results of backward stepwise multivariate Cox analysis in three models.

**Variables**	**Uni-Cox**		**BSR**		**LASSO**	
	**HR (95%CI)**	***P*** **value**	**HR (95%CI)**	***P*** **value**	**HR (95%CI)**	***P*** **value**
Age	1.04 (1.03–1.05)	< 0.001	1.04 (1.03–1.05)	< 0.001	1.04 (1.03–1.04)	< 0.001
Temperature	0.80 (0.74–0.86)	< 0.001	0.79 (0.73–0.84)	< 0.001	0.83 (0.78–0.89)	< 0.001
Hemoglobin	0.91 (0.85–0.99)	0.026				
PT	1.02 (1.00–1.03)	0.027				
Sodium	1.07 (1.03–1.11)	< 0.001	1.07 (1.03–1.11)	< 0.001		
Bicarbonate	0.95 (0.91–0.99)	0.012				
MV	2.86 (1.80–4.57)	< 0.001	2.85 (1.80–4.51)	< 0.001	3.57 (2.28–5.58)	< 0.001
Vasoactive agent	1.77 (1.25–2.49)	0.001	2.10 (1.50–2.94)	< 0.001	2.27 (1.63–3.17)	< 0.001
Furosemide	1.67 (0.97–2.88)	0.065				
Mannitol	3.19 (2.19–4.66)	< 0.001	3.01 (2.07–4.36)	< 0.001		
Liver cirrhosis	2.50 (1.24–5.04)	0.010	3.05 (1.53–6.06)	0.002		
Base of skull	0.74 (0.54–1.02)	0.066				
Extradural hematoma	1.82 (1.32–2.52)	< 0.001				
Loss of consciousness	2.29 (1.56–3.38)	< 0.001	2.16 (1.48–3.14)	< 0.001		

Uni-Cox, univariate Cox regression; BSR, best subset regression; LASSO, least absolute shrinkage and selection operator; HR, hazard ratio; CI, confidence interval; PT, prothrombin time; MV, mechanical ventilation.

### Evaluation and validation of the nomogram

**Figure 7:**
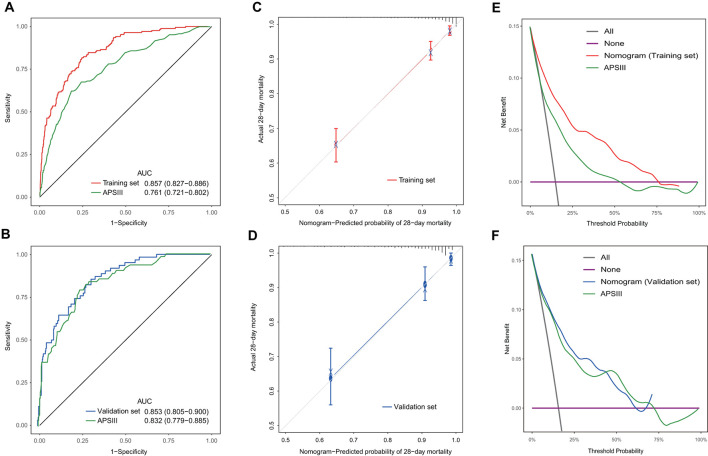
Discrimination, calibration, and clinical usefulness of the nomogram in the training and validation sets. **(A)** ROC curves of the nomogram and APSIII in the training set. **(B)** ROC curves of the nomogram and APSIII in the external validation set. **(C)** Calibration curve of the nomogram in the training set. **(D)** Calibration curve of the nomogram in the external validation set. **(E)** Decision curve analysis of the nomogram and APSIII in the training set. **(F)** Decision curve analysis of the nomogram and APSIII in the external validation set.

The VIF values for the mentioned variables were all below 1.5, indicating the absence of collinearity in our model. Our nomogram demonstrated better accuracy than APSIII for predicting the mortality at 28 days in the training set [0.857 (95% CI: 0.827–0.86) vs. 0.761 (95% CI: 0.721–0.802), DeLong's test: *P* < 0.001]. However, in the external validation set, no statistical significance existed [0.853 (95% CI: 0.805–0.900) vs. 0.832 (95% CI: 0.779–0.885), DeLong's test: *P* = 0.521] (Figures 7A, B). The C-index for the training set was 0.832 (95% CI: 0.765–0.883), and for the external validation set, it was 0.829 (95% CI: 0.712–0.905). After 10-fold cross internal validation, this model yielded an AUC value of 0.847 (95% CI: 0.846–0.848) and a C-index of 0.827 (95% CI: 0.826–0.827). Figures 7C, D displayed the calibration curve for the nomogram and it showed good calibration with a low Brier score of 0.091 (95% CI: 0.079–0.103) in the training set and 0.093 (95% CI: 0.074–0.111) in the external validation set, indicating the nomogram-predicted probability was highly consistent with the actual probability. The DCA curves (Figures 7E, F) revealed that the nomogram provided more net benefit than APSIII.

### Risk stratification based on the nomogram

Patients were categorized into low, mid, and high-risk groups based on total points calculated using the nomogram. Those with total points below 166.1 were classified as low-risk, while those with scores between 166.1 and 191.1 were considered as mid-risk. Those exceeding 191.1 points were allocated to the high-risk group (Supplementary Figure S3). Kaplan-Meier survival curves indicated that higher points were associated with lower survival probabilities (log-rank test: *P* < 0.001) (Supplementary Figure S4). The cut-off points for risk stratification demonstrated similar discrimination in the training set and the external validation set.

## Discussion

Skull fracture is a complex condition that not only disrupts the continuity of cranial bones but is also closely linked to various brain injuries. Those injuries can lead to symptoms ranging from mild alterations in consciousness to severe unconsciousness and even death. In the most severe cases, diffuse damage and swelling can affect the entire brain (11). In the UK, traumatic brain injury (TBI) stands as a leading cause of mortality and disability in individuals under 40 years old, presenting a significant public health challenge (12). Skull fracture, as a clinical event that is relatively easier to diagnose, provide clinicians with a more direct point for assessment and intervention. In emergency medical contexts, the utilization of our model can assist doctors in optimizing resource allocation and enhancing patient management efficiency. The prediction of fatal skull fractures is a field that has not been extensively researched, and our study contributes additional insights to the comprehension and management of skull fractures. In this retrospective study, we evaluated the records of 1,527 adult patients diagnosed with skull fracture from two databases. Nine predictive factors (age, temperature, sodium, MV, vasoactive agent, mannitol, extradural hematoma, loss of consciousness and GCS score) were selected to craft this nomogram. Our findings indicated that age, body temperature, and serum sodium levels were the top three features, carrying the most substantial weight.

Age plays a crucial role in determining the prognosis of various diseases (13–15). After adjusting for multiple factors in our analysis, older age was found to be an independent prognostic factor, exerting a relatively strong influence in the present nomogram. Growing evidence suggests that aging is associated with a decline in the body's immune function, known as “immunosenescence” (16). This weakened immune function can lead to a diminished capacity to defend against infections, which is a significant contributor to mortality in brain trauma patients (17, 18). Additionally, aging increase the vulnerability of microvessels, raising the risk of intracranial hemorrhage after skull fracture (19). Progressive brain atrophy, a characteristic of aging, leads to decreased brain tissue elasticity, increasing the risk of injuries such as diffuse axonal injury, acute subdural hematoma, and others after brain trauma (20, 21). Elderly patients also tend to have more comorbidities, further worsening their prognosis (22). For older patients, it is crucial to be vigilant about their heightened risk of mortality. Early detection and intervention in age-related health issues are key in improving outcomes for these patients.

There is inconsistency in the existing research findings regarding the impact of temperature on the prognosis of TBI patients. Some theories posit that hyperthermia, by increasing vascular permeability and promoting edema and inflammation, can lead to secondary brain injury in TBI cases. Conversely, lower temperature may slow down post-injury oxidative stress, cellular apoptosis, and inflammatory responses, providing neuroprotective effects for TBI patients (23). However, results from a phase III randomized trial showed no significant improvement in the prognosis of severe pediatric TBI with clinically induced hypothermia (24). Our nomogram highlights that body temperature carries significant weight, and as it decreased, the patient's prognosis worsens. There were several reasons that may be responsible for our findings. One possible explanation was that when body temperature drops, it can cause vasoconstriction and bradycardia, affecting the perfusion and oxygen delivery to vital organs (25). Another reason was perhaps that hypothermia can result in coagulation abnormalities, heightening the risk of bleeding (26). Therefore, we recommend that patients with skull fracture should be closely monitored for body temperature, and further exploration on this issue for such patients is deemed necessary.

Nearly 27% of critically ill patients suffer from various extents of hypernatremia during their hospitalization in the ICU (27). Our findings indicated that increased serum sodium levels were associated with a higher risk of death in patients with skull fractures. Hypernatremia has been proven in medical research to be closely related to poor prognosis. In a study involving COVID-19 patients, patients with hypernatremia had a 2.34–3.05 times higher risk of death compared to those with normal sodium levels (28). Additionally, in a study of cancer patients, patients with hypernatremia had significantly shorter survival times compared to those with normal sodium levels, and their hospital stays were also significantly longer (29). This electrolyte disorder not only reflects an impairment of the body's water balance but can also lead to serious neurological symptoms in patients. Causes of hypernatremia in patients with neurological disorders include the use of hyperosmotic fluids, limited access to free water, or conditions such as diabetes insipidus (30). Hu et al. found that hypernatremia was an independent prognostic factor for critically neurological patients (odds ratio: 1.192, 95% CI: 1.135–1.252, *P* < 0.001) (31). Cho et al. (32) found that hypernatremia had a strong independent association with poor long-term neurological outcomes in survivors of cardiac arrest.

Intracranial hemorrhage plays a pivotal role in evaluating the severity of head trauma. Research indicates that skull fracture is an independent risk factor for intracranial hemorrhage, which can negatively impact the neurological prognosis of patients with TBI (33). Extradural hemorrhage is the most common subtype of hemorrhage occurring in underage patients after skull fractures (34, 35). Skull fracture can tear blood vessels near the skull, leading to extradural hematoma (36). Our results indicated that extradural hematoma significantly influences the prognosis of skull fracture patients, causing brain tissue compression, increased intracranial pressure, and the risk of neural structure injury (37). This finding highlights the critical need for immediate identification of extradural hematoma in these patients. Understanding the grave implications of extradural hematoma in skull fractures enables clinicians to allocate resources more effectively and improve patient outcomes through targeted interventions.

In the management of brain injury patients, preventing hypoxia and hypotension is paramount. Mechanical ventilation and vasoactive agents are often used for this purpose. Our nomogram revealed that mechanical ventilation and vasoactive agents use were associated with higher mortality. In the ICU, patients who require mechanical ventilation and vasoactive drugs from the first day tend to have more severe conditions compared to those who do not need these interventions. The most common site of infection in patients with TBI admitted to ICU is respiratory system (38). Mechanical ventilation is considered a risk factor for ventilator-associated pneumonia, and the risk for TBI patients is approximately 42% (39). In order to achieve blood pressure and cerebral perfusion pressure (CPP) targets, we administer vasoactive drugs, such as norepinephrine (a widely used vasopressor in the worldwide). However, there is evidence pointing to its potential to induce vasospasm after intracranial hemorrhage, leading to a decrease in cerebral oxygenation (40). It's important to note that the association between vasoactive drugs use and increased mortality does not imply that these drugs are fatal. They are essential in specific circumstances, but their use should be carefully considered and monitored to ensure the best treatment outcomes and patient safety. Further research is needed to explore the relationship and potential mechanisms between vasoactive drugs and the prognosis of patients with skull fracture.

Increased intracranial pressure (ICP) is strongly linked to poor neurological outcomes and mortality in acute TBI patients (41). In clinical practice, mannitol is routinely employed to address elevated intracranial pressure. Nonetheless, in certain studies related to TBI, the early use of mannitol was found to be independently associated with a higher occurrence of AKI (42). Mannitol's diuretic action may lead to hypovolemia and hypoperfusion, posing a significant risk for increased morbidity and mortality in patients with brain pathology (43). Our analysis showed that mannitol use was associated with poor patient outcomes. Further studies are needed to investigate the mechanisms behind this association and explore other factors that may influence the correlation between mannitol usage and mortality risk. Finally, our analysis also revealed that the loss of consciousness contributed to an augmented risk of 28-day mortality. The occurrence of consciousness loss in these patients may indicate more severe brain damage and consequent neurological dysfunction. This severe neurological dysfunction may subject them to a greater incidence of complications and risks during their hospitalization, ultimately raising the probability of death.

In this study, by employing univariate Cox regression, BSR, LASSO, and backward stepwise multivariable Cox regression, our analysis effectively reduced the risk of overfitting and underfitting. Moreover, the availability of all variables in the final model guarantees the practicality and clinical utility of this nomogram. However, there are limitations to consider. Not all patients' diagnostic information in the public databases was consistently well-defined, impacting the precision of our results, particularly in cases with ambiguous diagnoses like “unspecified” fracture types. Additionally, the limited data in the databases result in the exclusion of critical factors that could impact patients' survival, such as detailed information on injury causes and processes, which could have informed fracture site analysis and severity assessment. For issues with inconsistent diagnostic information, we can build separate models for patients with different diagnoses in the future. For ambiguous diagnosis issues, we can try to exclude these patients during data preprocessing. For the challenge of limited data, developing models based on large datasets from multiple centers is a viable approach. We believe that implementing these measures will enhance the stability and generalizability of our model.

## Conclusion

In summary, we have successfully developed and validated the first prognostic model that integrates nine clinical features to effectively predict the 28-day mortality risk in ICU patients with skull fractures. Early intervention in modifiable variables in the model, such as body temperature and serum sodium, can significantly improve the prognosis in skull fracture patients. Previous studies relating to skull fracture emphasized risk factors but didn't combine them into a full model. Our research addressed this gap by developing a comprehensive model that integrates several variables, potentially aiding clinicians in making well-informed decisions regarding the management of skull fractures.

## Data availability statement

The raw data supporting the conclusions of this article will be made available by the authors, without undue reservation.

## Author contributions

JT: Writing—original draft. ZZ: Writing—review & editing. MN: Writing—review & editing. CW: Writing—review & editing.
