# Supplementary material for: Establishment and external validation of a nomogram for predicting 28-day mortality in patients with skull fracture
Source: Front Neurol. 2024 Jan 12;14:1338545. doi: 10.3389/fneur.2023.1338545 (PMC10811263; doi:10.3389/fneur.2023.1338545)

**Table S1** SQL queries for the selection of patients with skull fracture.

| **Cohort** | **SQL queries** |
| --- | --- |
| MIMIC | SELECT * FROM mimiciv_hosp.d_icd_diagnoses d WHERE  ((d.long_title LIKE '%skull%' and d.long_title LIKE '%fracture%')  or (d.long_title LIKE '%frontal%' and d.long_title LIKE '%fracture%')  or (d.long_title LIKE '%parietal%' and d.long_title LIKE '%fracture%')  or (d.long_title LIKE '%occiput%' and d.long_title LIKE '%fracture%')  or (d.long_title LIKE '%occipital%' and d.long_title LIKE '%fracture%')  or (d.long_title LIKE '%temporal%' and d.long_title LIKE '%fracture%')  or (d.long_title LIKE '%sphenoid%' and d.long_title LIKE '%fracture%')  or (d.long_title LIKE '%ethmoid%' and d.long_title LIKE '%fracture%')  and d.long_title NOT LIKE '%without mention of skull fracture%') |
| eICU | SELECT * FROM diagnosis WHERE diagnosisstring like '%skull%'  and diagnosisstring like '%fracture%' |

Abbreviations: SQL, Structured Query Language; MIMIC, Medical Information Mart for Intensive Care.

**Figure S1**. Missing values in the training set.


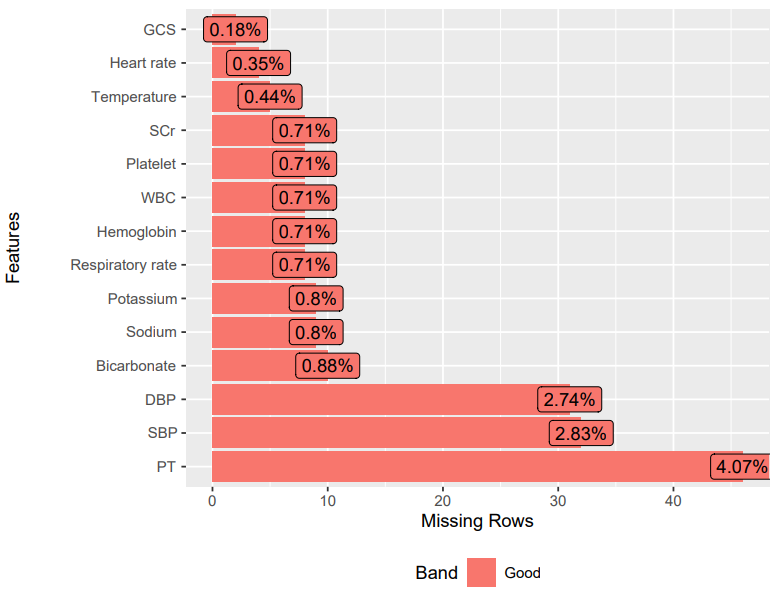


**Figure S2**. Missing values in the external validation set.


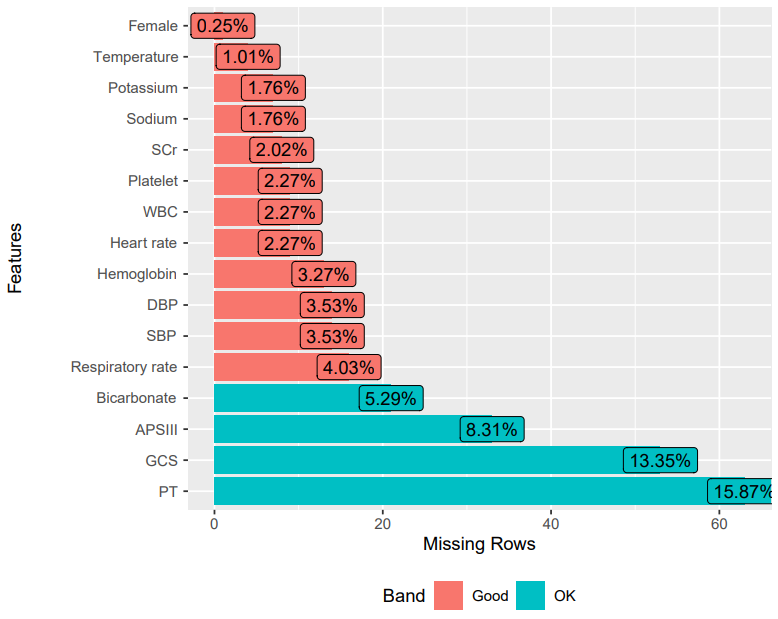


**Table S2** Comparison of variables in the training set before and after multiple imputation.

| **Variables** | **Before imputation** | **After imputation** | **P value** |
| --- | --- | --- | --- |
| GCS (point) | 13.00 [9.00, 15.00] | 13.00 [9.00, 15.00] | 0.997 |
| Heart rate, bpm | 86.00 [73.00, 99.00] | 86.00 [73.00, 99.00] | 0.986 |
| Temperature, ℃ | 36.83 [36.33, 37.22] | 36.83 [36.33, 37.22] | 0.959 |
| SCr, mg/dL | 0.90 [0.70, 1.10] | 0.90 [0.70, 1.10] | 0.978 |
| WBC, 10^9^/L | 13.30 [9.80, 17.80] | 13.30 [9.80, 17.80] | 0.916 |
| Platelet, 10^9^/L | 226.00 [182.00, 280.00] | 226.00 [182.00, 280.00] | 0.969 |
| Hb, g/dL | 12.90 [11.40, 14.20] | 12.90 [11.40, 14.20] | 0.995 |
| Respiratory rate, bpm | 18.00 [15.00, 21.00] | 18.00 [15.00, 21.00] | 0.959 |
| Sodium, mmol/L | 140.00 [137.00, 142.00] | 140.00 [137.00, 142.00] | 0.940 |
| Potassium, mmol/L | 3.90 [3.60, 4.30] | 3.90 [3.60, 4.30] | 0.979 |
| Bicarbonate, mmol/L | 23.00 [20.00, 25.00] | 23.00 [20.00, 25.00] | 0.986 |
| DBP, mmHg | 70.00 [60.00, 81.00] | 70.00 [60.00, 81.00] | 0.995 |
| SBP, mmHg | 130.00 [115.00, 144.00] | 131.00 [114.00, 144.00] | 0.988 |
| PT | 12.60 [11.60, 13.50] | 12.60 [11.60, 13.50] | 0.955 |

Abbreviations: GCS, Glasgow Coma Scale; SCr, serum creatinine; WBC, white blood cell count; Hb, hemoglobin; DBP, diastolic blood pressure; SBP, systolic blood pressure; PT, prothrombin time.

**Table S3** Comparison of variables in the external validation set before and after multiple imputation.

| **Variables** | **Before imputation** | **After imputation** | **P value** |
| --- | --- | --- | --- |
| Female, % | 94 (23.7) | 94 (23.7) | 1.000 |
| Temperature, ℃ | 36.70 [36.28, 37.00] | 36.70 [36.28, 37.00] | 0.998 |
| Potassium, mmol/L | 3.80 [3.50, 4.20] | 3.80 [3.50, 4.20] | 0.886 |
| SCr, mg/dL | 0.90 [0.73, 1.10] | 0.90 [0.73, 1.10] | 0.970 |
| Sodium, mmol/L | 139.00 [137.00, 141.00] | 139.00 [137.00, 141.00] | 0.906 |
| WBC, 10^9^/L | 12.85 [9.30, 17.00] | 12.80 [9.30, 17.00] | 0.972 |
| Heart rate, bpm | 86.00 [71.00, 102.00] | 86.00 [71.00, 102.00] | 0.973 |
| Hb, g/dL | 13.65 [12.10, 14.80] | 13.60 [12.10, 14.80] | 0.937 |
| Platelet, 10^9^/L | 224.00 [182.00, 264.25] | 223.00 [182.00, 264.00] | 0.891 |
| DBP, mmHg | 77.00 [65.00, 88.00] | 77.00 [65.00, 89.00] | 0.968 |
| SBP, mmHg | 134.00 [116.00, 148.50] | 134.00 [116.00, 148.00] | 0.948 |
| Respiratory rate, bpm | 18.00 [16.00, 21.00] | 18.00 [16.00, 21.00] | 0.882 |
| Bicarbonate, mmol/L | 23.00 [20.75, 26.00] | 23.00 [21.00, 26.00] | 0.622 |
| APSIII (point) | 41.50 [28.00, 69.25] | 42.00 [28.00, 70.00] | 0.952 |
| PT, s | 13.35 [12.00, 14.50] | 13.50 [12.20, 14.50] | 0.862 |
| GCS (point) | 8.00 [3.00, 14.00] | 8.00 [3.00, 14.00] | 0.834 |

Abbreviations: SCr, serum creatinine; WBC, white blood cell count; Hb, hemoglobin; SBP, systolic blood pressure; DBP, diastolic blood pressure; APSIII, acute physiology score III; PT, prothrombin time; GCS, Glasgow Coma Scale.

**Table S4** Receiver operating characteristic curve analysis of three models.

| **Values** | **Uni-Cox** | **BSR** | **LASSO** |
| --- | --- | --- | --- |
| AUC (95% CI) | 0.857 (0.827-0.886) | 0.861 (0.832-0.889) | 0.838 (0.807-0.868) |
| P value | <0.001 | <0.001 | <0.001 |
| Se (%) | 81.5 | 76.8 | 76.2 |
| Sp (%) | 74.8 | 80.4 | 79.8 |
| PPV (%) | 36.1 | 40.6 | 39.8 |
| NPV (%) | 95.9 | 95.2 | 95.0 |
| Cut-off | 80.484 | 49.027 | 86.785 |

Abbreviations: Uni-Cox, univariate Cox regression; BSR, best subset regression; LASSO, least absolute shrinkage and selection operator; AUC, area under the curve; CI, confidence intervals; Se, sensitivity; Sp, specificity; PPV, positive predictive value; NPV, negative predictive value.

**Figure S3.** Risk stratification results presented with X-tile software.


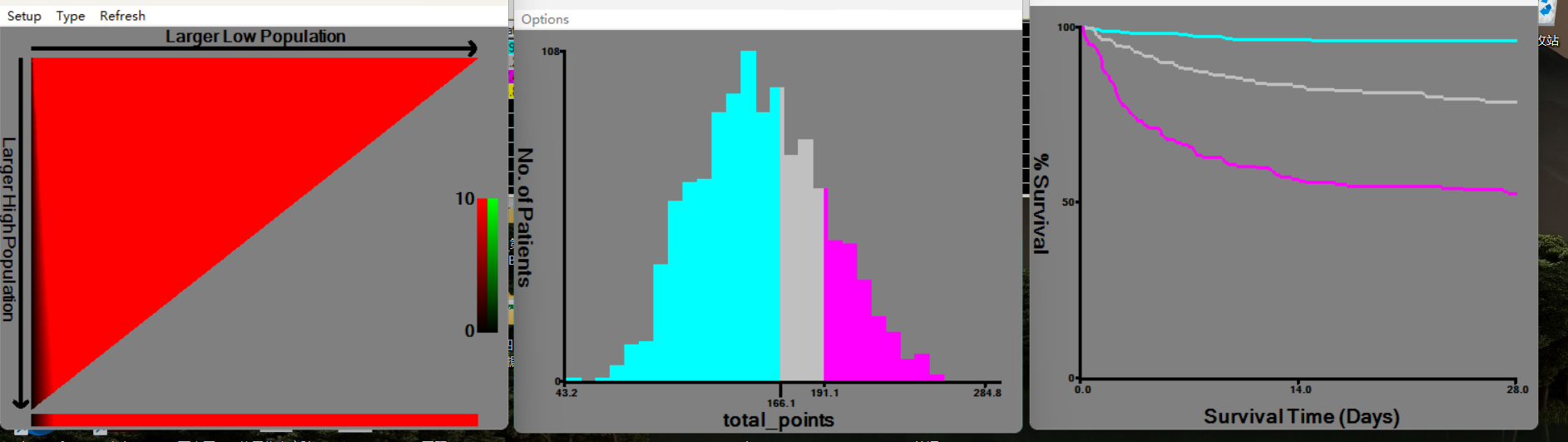

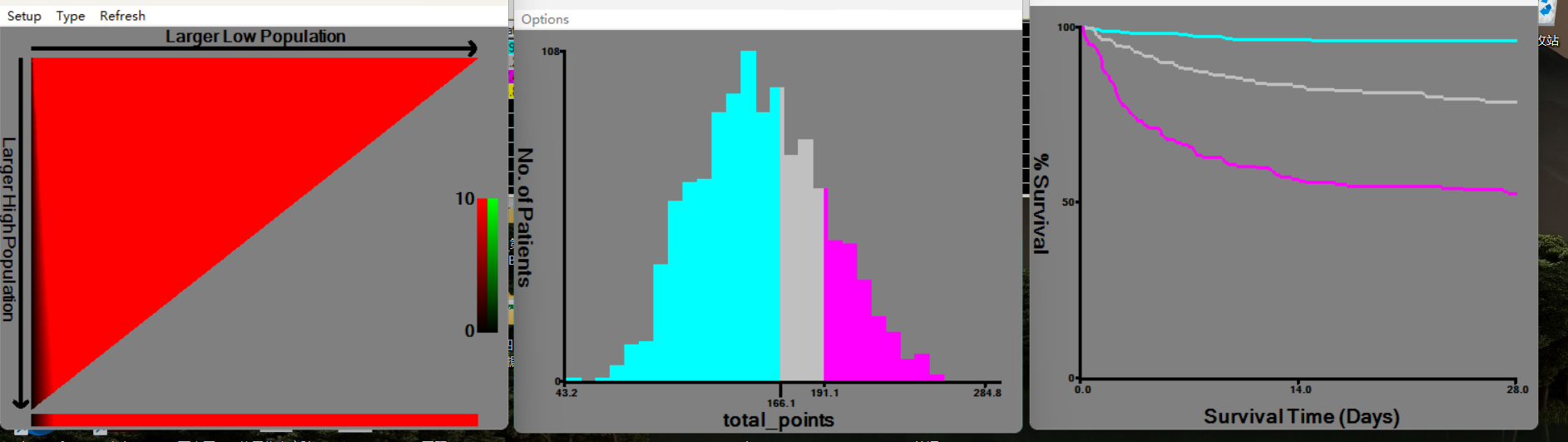

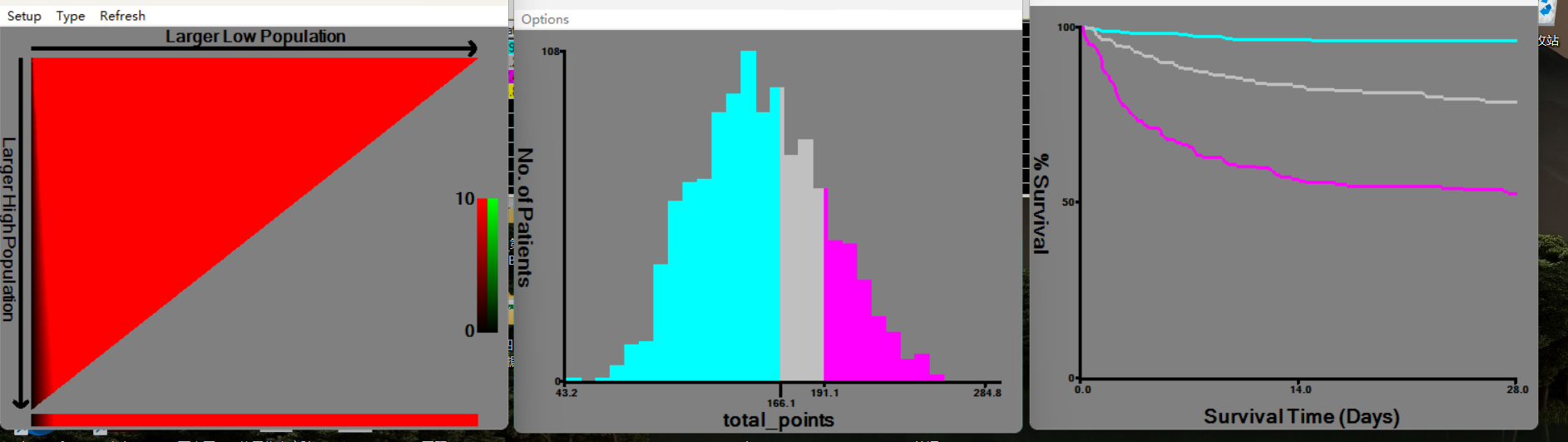

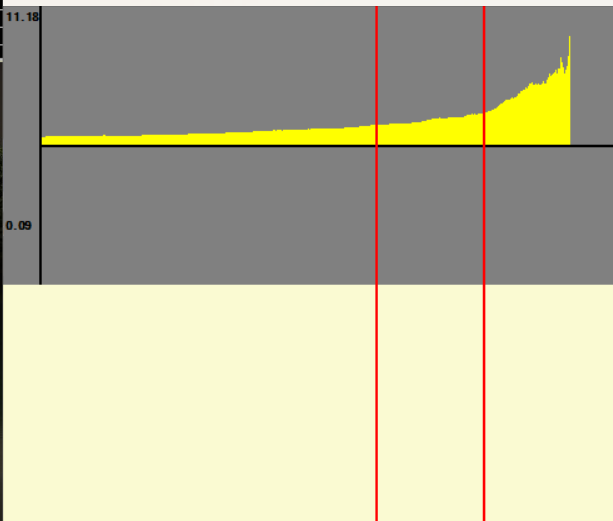


**Figure S4** Kaplan-Meier curves of patients grouped by nomogram-based risk stratification. (A) Training set. (B) External validation set.


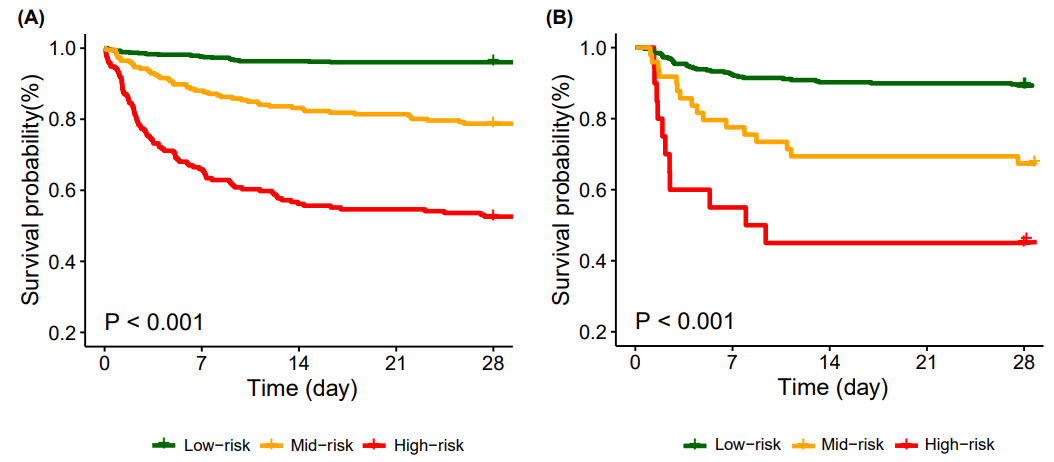

Supplement: Supplementary file 1 [file Data_Sheet_1.docx]
